# The value of FDG-PET/CT imaging in the assessment, monitoring, and management of COVID-19

**DOI:** 10.1140/epjp/s13360-023-03797-6

**Published:** 2023-03-27

**Authors:** Matthew T. Griffin, Thomas J. Werner, Abass Alavi, Mona-Elisabeth Revheim

**Affiliations:** 1 Drexel University College of Medicine, Philadelphia, PA USA; 2 Department of Radiology, University of Pennsylvania, Philadelphia, PA USA; 3 Division of Radiology and Nuclear Medicine, Oslo University Hospital, Postbox 4950, 0424 Nydalen, Oslo, Norway; 4 Faculty of Medicine, Institute of Clinical Medicine, University of Oslo, Postbox 1078, 0316 Blindern, Oslo, Norway; 5 The Intervention Center, Division of Technology and Innovation, Oslo University Hospital, Postbox 4950, 0424 Oslo, Norway

## Abstract

The pathogenesis of Coronavirus Disease 2019 (COVID-19) involves cytokine-driven recruitment and accumulation of inflammatory cells at sites of infection. These activated neutrophils, monocytes, and effector T cells are highly glycolytic and thus appear as [18]F-labeled fluorodeoxyglucose (FDG) avid sites on positron emission tomography (PET) imaging. FDG-PET-computed tomography (FDG-PET/CT) is a highly sensitive modality for the detection, monitoring, and assessing response related to COVID-19 disease activity that holds significant clinical relevance. To date, concerns over cost, access, and undue radiation exposure have limited the use of FDG-PET/CT in COVID-19 to a small number of individuals where PET-based interventions were already indicated. In this review, we summarize the existing literature on the use of FDG-PET in the detection and monitoring of COVID-19 with particular focus on several areas of clinical relevance that warrant future research: (1) incidental early detection of subclinical COVID-19 in patients who have undergone FDG-PET for other underlying diseases, (2) standardized quantitative assessment of COVID-19 disease burden at specific points in time, and (3) analysis of FDG-PET/CT data leading to better characterization of COVID-19 pathogenesis. Employing FDG-PET/CT for these purposes may allow for the earliest detection of COVID-19-associated venous thromboembolism (VTE), standardized monitoring of disease progression and response to treatment, and better characterization of the acute and chronic complications of this disease.

## Introduction

Coronavirus disease 2019 is an infectious disease driven by the spread of the novel SARS-CoV-2 β-coronavirus. COVID-19 primarily involves the lungs and is associated with upper respiratory tract infection in mild cases and fulminant pneumonia in severe disease [1, 2]*.* Although best known for its pulmonary presentations, COVID-19 can also manifest systemically and can lead to inflammation in other organs including the brain, bowel, liver, kidney, and cardiovascular system [2–6]. Fever, cough, and shortness of breath are the most common symptoms followed by malaise, body ache, abdominal pain, diarrhea, sputum production, and loss of taste and smell [1]. Elevated acute phase reactants, including ferritin, D-dimers, and pro-inflammatory cytokines, have been associated with severe pathologic states [2, 4, 7]. Patients with COVID infection are prone to developing cerebrovascular complications including deep vein thrombosis leading to pulmonary embolism [8]*.* Additionally, a significant proportion of patients who have had acute COVID-19 develop persistent symptoms and a range of sequelae affecting the kidneys, lungs, heart, and brain [9–12]. Reverse transcription polymerase chain reaction (RT-PCR) is frequently employed for identification of SARS-CoV-2 infection, while a variety of blood tests and imaging techniques are typically performed to evaluate the extent, severity, and associated complications of the disease [2, 3, 13]*.* Chest X-ray, chest computed tomography (CT), magnetic resonance imaging (MRI), and positron emission tomography (PET), and, recently, point-of-care ultrasound (PUS) have been employed to detect and evaluate various inflammatory lesions [2, 3]*.* With new COVID-19 variants emerging and over 600 million confirmed cases to date, there is an urgent need for improved assessment, monitoring, and management of COVID-19 infection.

The appropriate roles of the various radiologic imaging modalities in the initial diagnosis and monitoring of COVID patients have been the subject of intense debate [2, 4, 14–16]. Concerns over undue radiation exposure and low specificity led many professional societies and expert panels to issue recommendations against the use of chest imaging as a primary screening modality [2, 14, 16]. Nevertheless, radiologic imaging techniques continue to play a central role in the staging and prioritizing of COVID patients, particularly in resource limited triage situations [14, 16–18]. Furthermore, the need to characterize the systemic manifestations of COVID-19, assess disease burden at different points in time, and evaluate the efficacy of potential treatments requires the continued use of imaging techniques [2, 3, 19, 20]*.* With these goals in mind, several groups have set out to summarize the characteristic findings associated with COVID-19 as revealed by various imaging modalities and present their views about the potential value of each technique [2, 3, 14–17, 21, 22]*.*

Recently, Fields et al*.* published a review with particular focus on CT, MRI, and PET in COVID-19 [2]. Despite its low specificity and qualitative nature, the affordability, availability, and detailed anatomical resolution of chest CT have made it the modality of choice for much of the workup and staging of COVID-19, particularly when evaluating complications and progression of the disease [2, 3, 14]. Classic features of COVID-19 infection on chest CT include multifocal peripheral ground-glass opacities, with or without peripheral consolidations, in a bilateral, peripheral, and posterior distribution [14, 18]. Recently, MRI has shown excellent concordance with chest CT and has been proposed as a viable alternative for monitoring COVID-19 pneumonia in patients in whom exposure to ionizing radiation should be avoided such as children and pregnant patients [2, 23, 24]. PUS has also been proposed as an alternative technique for examining pulmonary lesions in these populations although more research is required to investigate its effectiveness in distinguishing between different stages of parenchymal infiltration compared to CT [25]*.* While each of these structural imaging techniques, CT, US, MRI, and conventional radiography, has been employed in the diagnosis and monitoring of COVID-19 in particular patient populations, an in-depth discussion of the merits, limitations, and disadvantages of each technique is beyond the scope of this review. Rather, we aim to summarize the existing literature on the use of FDG-PET in the detection and monitoring of COVID-19 with particular focus on several areas of potential clinical application that warrant additional attention and research: (1) incidental early detection of subclinical COVID-19 in patients where FDG-PET is already clinically indicated, (2) standardized quantitative assessment of COVID-19 disease burden at specific points in time, and (3) analysis of FDG-PET/CT data leading to better characterization of COVID-19 pathogenesis and its acute and chronic complications.

## FDG-PET

[18]F-labeled fluorodeoxyglucose (FDG) is a positron-emitting glucose analogue that allows for the imaging and measurement of glucose metabolism throughout the body with PET*.* FDG-PET, used in conjunction with CT or MRI, has been extensively employed to assess numerous organ diseases and disorders [26–32]*.* Although best known for its widespread use in detecting, staging, and monitoring of various malignancies, the role of FDG-PET imaging in the detection and characterization of inflammatory disorders has been validated in many settings including chronic obstructive pulmonary disease (COPD), asthma, and interstitial lung disease (ILD) [33–37]. In the context of viral pneumonia, chemokine recruitment drives the migration of inflammatory cells to sites of infection. These activated neutrophils, monocytes, and effector T cells are highly glycolytic and thus manifest as FDG avid foci on PET imaging [2, 3, 19, 34, 35]*.* FDG avid foci highlight sites of infection while quantification of uptake using semi-quantitative parameters such as standardized uptake values (SUVs) serve to measure the extent of inflammatory activity in specific regions of the body [3, 19, 29–32, 34, 35, 38–43]

**Fig. 1:**
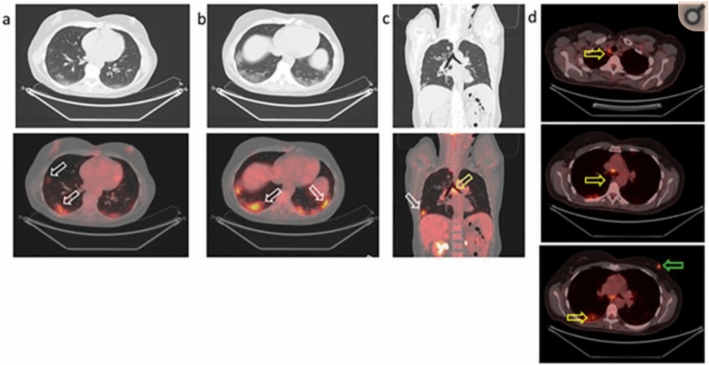
Positron Emission Tomography/Computed Tomography with [18]F-labeled fluorodeoxyglucose (FDG) to restage breast cancer in a 57-year-old woman. Increased Uptake of FDG (white arrows) in subpleural ground-glass opacities of both lungs in the axial (**a**,**b**) and coronal (**c**) section. Tracer uptake in the carinal (**c**) and mediastinal (**d**) lymph nodes due to infection (yellow arrows). Focal tracer uptake in the left breast corresponds to the suspected nodule (**d**; green arrow) (Reproduced from M. Scarlattei et al. [60] under creative commons use)

Although not recommended as a first-line investigative modality, FDG-PET/CT is highly sensitive in the detection of COVID-19-associated pulmonary lesions [2, 3, 20, 38, 44–46]*.* Numerous reports dating back to the first months of the pandemic have noted incidental localization of FDG uptake in the lungs of asymptomatic, later COVID-19 positive, patients who had undergone PET imaging for other clinical indications [45, 47–53] [Fig. 1—Illustrative figure of classical incidental findings and FDG uptake]. Following these reports and evidence that SARS-CoV-2 replication occurs in absence of clinical manifestations of disease, FDG-PET was proposed as an alternative method for detecting subclinical infection [2, 19, 21, 38]*.* This sensitivity combined with FDG-PET’s unique ability to quantify diffuse disease activity throughout the body suggests FDG-PET/CT may be our best tool for standardized COVID-19 monitoring, characterization of the systemic manifestations of COVID-19, and quantitative assessment of patients' response to treatment [3, 20, 38, 44, 54].

To date, valid concerns of cost, radiation burden, low specificity, and prolonged COVID-19 exposure times for imaging staff have limited the use of FDG-PET screening to a small number of patients in whom FDG-PET is clinically indicated [2, 21]. In the context of these concerns and limitations, we aim to summarize the existing literature on the use of FDG-PET in the detection and monitoring of COVID-19 with special focus on several areas that are relevant in the management of this infection.

### Incidental early detection of subclinical COVID-19 infection

FDG-PET/CT is most frequently employed for diagnosing and staging cancer, monitoring response to treatment, and detecting recurrence in various malignancies [55]. For these patients, and others who are already undergoing this modality, FDG-PET/CT imaging can provide vital information at the cellular level and may allow for the earliest detection of asymptomatic COVID-19 infection [5, 38, 46–51, 56, 57]. A systematic review and meta-analysis of 24,410 adults in 148 studies from nine countries, concluded that symptoms alone are not sufficient for diagnosis in a large proportion of SARS-CoV-2 infected individuals [1]. Several other groups noted significantly increased detection rates for interstitial pneumonia-associated pulmonary lesions in patients with cancer undergoing FDG-PET during the pandemic*,* some of which led to the initial COVID-19 diagnosis [45, 47–50, 56, 58, 59]. Particularly for patients in immunocompromised states, early detection and prompt initiation of supportive care is crucial for improving outcomes and maximizing survival [2, 5, 21, 49]. These factors led several groups to call attention to the potential clinical utility of FDG-PET in the differential diagnosis of complex, suspected COVID-19 cases [19, 39, 45, 47, 48, 51, 57, 60].

**Fig. 2:**
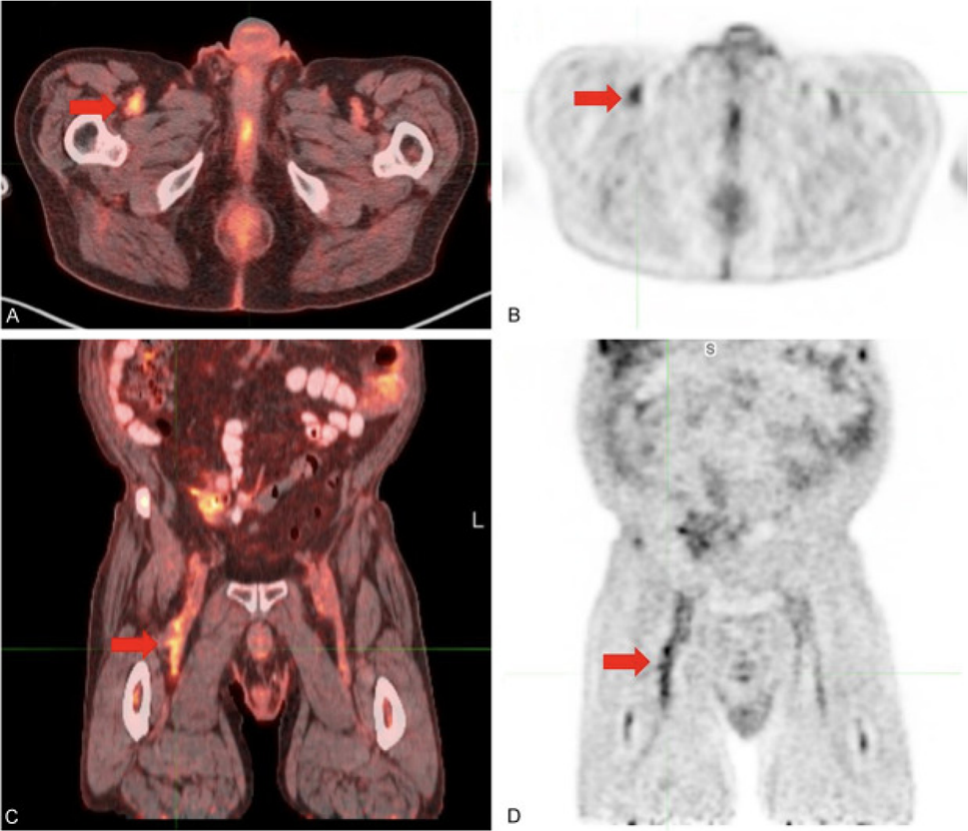
Venous thrombosis in the right common femoral vein, diagnosed prior to the ultrasound. **A** Axial FDG-PET/CT, **B** axial FDG-PET, **C** coronal FDG-PET/CT, and **D** coronal FDG-PET demonstrated high metabolic activity in the lumen of the right common femoral vein, consistent with venous thrombosis. Ultrasound performed two weeks later confirmed non-occlusive thrombus in the right common femoral vein (Reproduced from Kaghazchi et al. [69] under open copyright policy of the journal)

The ability for FDG-PET/CT to detect and localize venous thrombosis provides further justification for its use in COVID-19 diagnosis. Patients with COVID-19 are prone to venous thrombosis which can lead to devastating cardiovascular complications including pulmonary embolism (PE) [8, 38, 61]. In a study by Klok et al*.* as many as 27% of COVID-19 positive patients at the intensive care unit were found to have venous thromboembolism (VTE), and of these, 81% were diagnosed with pulmonary embolism (PE) [62]. Based on the longstanding experience with this disease, the clinical signs and symptoms of acute PE are nonspecific. Some patients can be asymptomatic, and others may have symptoms overlapping with the symptoms of COVID-19 or its associated complications [63] making it difficult to diagnose VTE. Venous thrombi contain a high concentration of activated leukocytes and are readily visualized on FDG-PET [3, 38, 61, 64–67]. Prior research suggests that FDG-PET imaging can distinguish acute from chronic VTE and identify patients at high risk of VTE [64, 65]. Zhu et al*.* demonstrated a strong association between lower extremity venous uptake of FDG and VTE risk but suggested this association does not appear to be related to the location or timing of the VTE [64]. Kaghazchi et al*.* noted that FDG-PET/CT can assess deep structures like the inferior vena cava which are not accessible by traditional ultrasonography [67]. These findings have led several groups to propose the use of total-body FDG-PET/CT in the detection and characterization of VTE in high-risk patients [38, 61, 64, 65, 67, 68] [Fig. 2—FDG-PET/CT image with visible thrombi] [69]. In the context of suspected COVID patients or patients with cancer undergoing imaging procedures, total-body FDG-PET/CT imaging may allow for the earliest detection of VTE leading to more timely interventions and better outcomes [3]. While these data are readily available in this patient population, most physicians do not pay attention to venous uptakes when reporting FDG-PET/CT imaging [61]. Future studies and physician education initiatives are necessary to confirm and successfully employ this novel application of FDG-PET imaging.

### Quantitative assessment of systemic and organ-specific COVID-19 severity

One of the major advantages of FDG-PET is its ability to quantify diffuse disease activity in various organs throughout the body [3, 29–32, 38, 40–43]. To date, FDG-PET/CT imaging has been employed for the global assessment of inflammation in the entire lungs, atherosclerosis throughout the cardiovascular system, total-body tumor burden in patients with cancer, global assessment of musculoskeletal disorders, and systemic conditions such as sarcoidosis [39]. In the context of COVID patients, this technology may represent a powerful tool for assessing whole-body and organ-specific disease severity, characterizing the systemic manifestations of COVID-19, and monitoring response to potential treatments.

A single total-body FDG-PET scan provides a quantitative landscape of disease activity at the molecular level [28, 38, 41]*.* These data can be converted into a quick, highly reproducible, operator independent, AI based measurement of Global Disease Score (GDS) which is a single number representing the total body’s disease burden at that time [70]. In the context of systemic, heterogeneously presenting diseases like COVID-19, the GDS may allow for the standardized measurement and monitoring of a patient’s disease burden, progression, and response to therapeutic interventions [3, 38, 39, 70]. This capability has been validated in other settings and makes total-body FDG-PET exceptionally well-suited for the examination of patients with SARS-CoV-2 infection [3, 31, 38, 68, 70–73].

### FDG-PET/CT data analysis leading to better characterization of COVID-19 pathogenesis

**Fig. 3:**
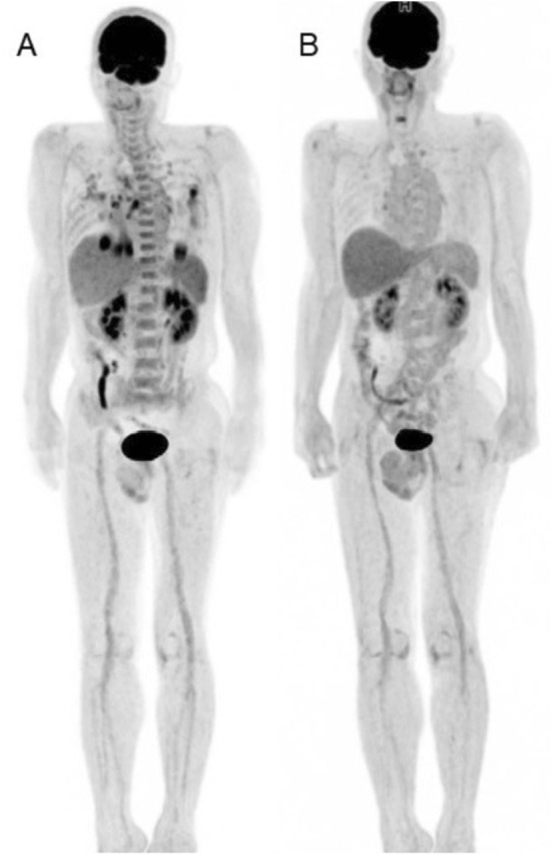
Whole-body [18]F-labeled fluorodeoxyglucose (FDG)-PET image of COVID-19. **A** Whole-body FDG-PET image of the patient with COVID-19 pneumonia (4 weeks after symptom onset and 3 weeks after negative RT-PCRs). **B** Whole-body FDG-PET image of the patient with COVID-19 pneumonia (4 weeks from previous FDG-PET scan). Intense FDG uptake was confirmed in lung lesion and mediastinal lymph node in the first image. In addition, increase FDG was seen in bone marrow and spleen. FDG uptake in the lung lesion and mediastinal lymph node disappeared, and uptake in the bone marrow and spleen decreases as physiological uptake level (Reproduced from Minamimoto et al*.* [54] under open copyright policy of the journal)

**Fig. 4:**
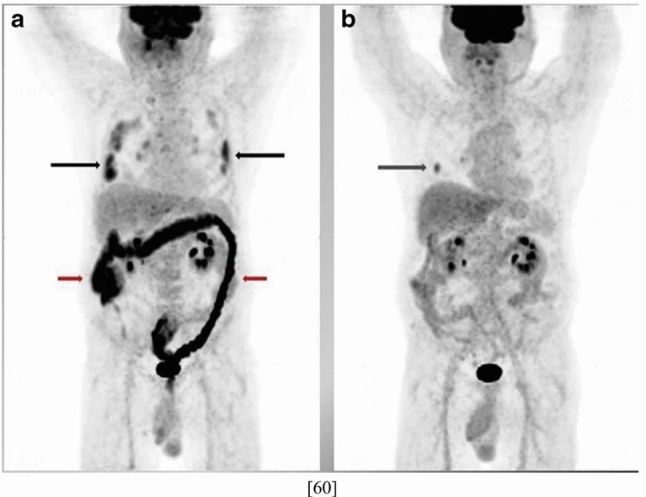
**a** The initial [18]F-labeled fluorodeoxyglucose PET/CT study reveals metabolically active lung infiltrates (black arrows), hypermetabolic hilar and mediastinal lymphadenopathy, and intense colonic activity (red arrows). **b** Follow-up PET/CT with resolution of lung and GI activity, leaving a metabolically active nodule that was obscured on the initial PET-CT by hypermetabolic COVID-19 pulmonary infiltrate (arrow). *CT* computed tomography, *GI* gastrointestinal, *PET* positron emission tomography (Reproduced with permission from Kidane et al. [59])

**Fig. 5:**
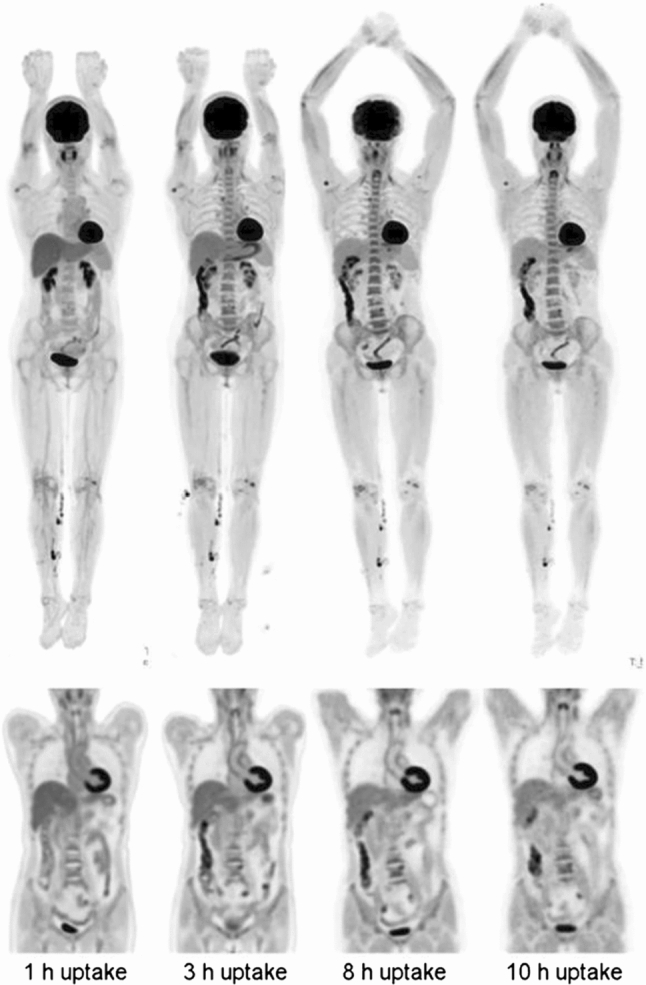
Delayed imaging for subject (256 MBq injected, 14 min scan duration). (Left-to-right) Images from scans performed at 1, 3, 8, and 10 h after injection. (Top row) MIP images. (Bottom row) coronal views of thorax and abdomen. Head motion artifacts are visible in 8-h scan (This research was originally published in JNM. Badawi et al. [71]^©^ SNMMI)

**Fig. 6:**
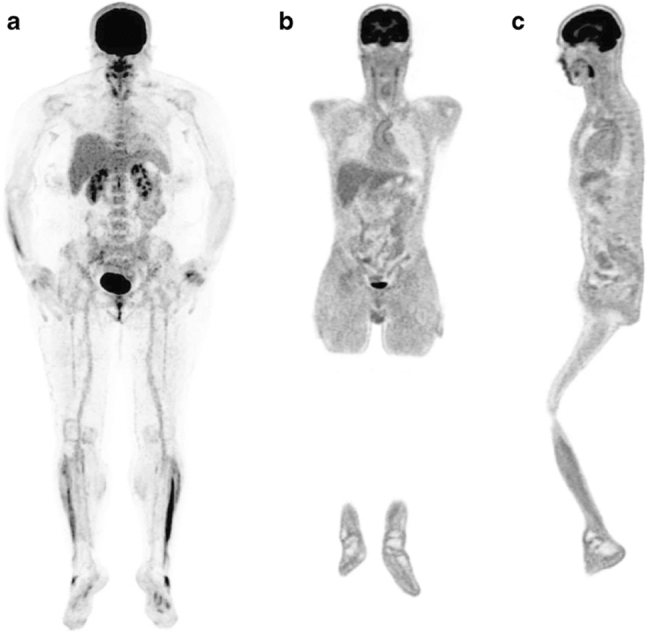
FDG-PET/CT in recovered SARS-CoV-2 patient. Example of [18]F-labeled fluorodeoxyglucose (FDG) bone marrow uptake in a 50-year-old female patient with persisting symptoms (dypsnea and fatigue) lasting almost 3 months (MIP in **a**). The patient took only symptomatic treatment during the acute phase of infection. Example of FDG vascular uptake in a 51-year-old male patient with persisting symptoms (fatigue) lasting for almost 4 months. He developed an acute respiratory distress syndrome, which finally required a tracheal intubation and invasive ventilation. Visually, the FDG uptake was scored as grade 2 at the ascending aorta, the aortic arch (**b**), and the descending aorta (**c**). (Reproduced with permission from Sollini et al. [75])

Total-body FDG-PET imaging may also hold substantial value as we work to characterize the systemic manifestations of COVID-19 [3, 6, 20, 34, 38, 39, 54, 74]*.* Preliminary evidence suggests that SARS-CoV-2 infection may induce vascular inflammation and that hyper-inflammation may magnify the symptomatic presentation of COVID-19 disease [3, 20, 75]. COVID-19-associated inflammation has been observed in organs outside the lungs including the brain, bowel, liver, kidney, and cardiovascular system though the mechanism underlying these presentations is not well-understood [2–6, 38] [Figs. 3, 4, 5, 6—COVID-associated inflammation outside the lungs].

**Fig. 7:**
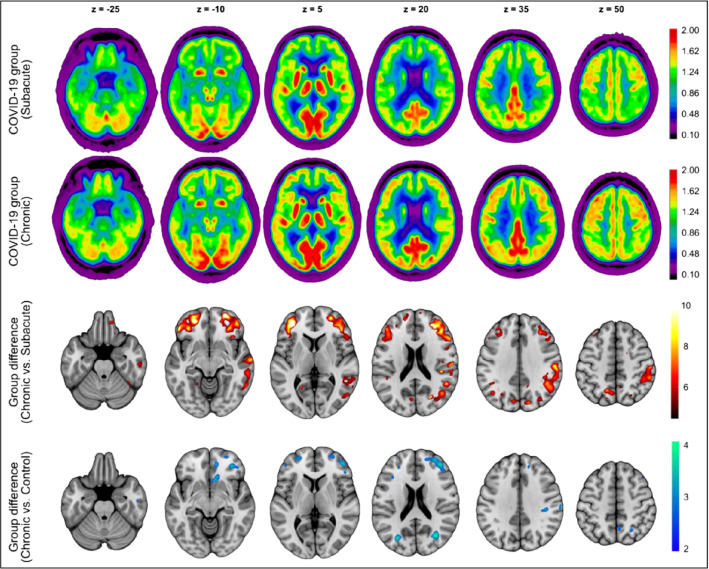
Result of [18]F-FDG-PET group analysis. (First and second rows) Transaxial sections of group-averaged spatially normalized [18]F-labeled fluorodeoxyglucose (FDG)-PET scans in COVID-19 patients at subacute and chronic stages (n = 8; initially requiring inpatient treatment for nonneurologic complications). (Third and fourth rows) Results of statistical parametric mapping analysis. Third row illustrates regions that show significant increases in normalized FDG uptake in COVID-19 patients at chronic stage compared with subacute stage (paired *t* test, *P* < 0.01, false-discovery rate-corrected). Fourth row depicts regions that still show significant decrease in normalized FDG uptake in chronic stage compared with age-matched control cohort (2-sample *t* test, *P* < 0.005). SPM12 *t* values are color-coded and overlaid onto MRI template. Images are presented in neurologic orientation, that is, left image side corresponds to the patients left body side; numbers denote axial (z) positions in millimeters. (This research was originally published in JNM. Blazhenets et al. [11]^©^ SNMMI)

Over the past decade, FDG-PET/CT imaging has emerged and demonstrated great utility for detecting and characterizing various infectious and inflammatory disorders including chronic osteomyelitis, tuberculosis, lower-limb protheses, complicated diabetic foot, acquired immunodeficiency syndrome (AIDS), common variable immunodeficiency (CVID), and vascular graft infection [29, 34–36, 38]. In each case, FDG-PET/CT provides a highly sensitive, quantitative portrait of the glycolytic activity of the cells associated with the inflammatory response [74, 76]. In contrast to FDG uptake in cancerous cells, the affinity of glucose transporters to deoxyglucose during inflammation appears to be enhanced by cytokines and growth factors, which is increased by cellular stress due to cell injury. These biological factors make FDG-PET ideally suited for investigating COVID-19-associated inflammation [3, 74–76]*.* Following this line of thought, Sollini et al*.* analyzed FDG-PET/CT imagery of 13 COVID-19 patients who complained of symptoms for more than 30 days compared to 26 COVID-19 negative melanoma patients in a prospective case–control study [76]*.* These investigators noted many organ/parenchyma uptake values (SUVs and SUV ratios) significantly differed between the two groups, and COVID-19 patients exhibited brain hypometabolism in the right parahippocampal gyrus and thalamus. These specific areas of hypometabolism in the brain were associated with persistent anosmia/ageusia, fatigue and vascular uptake [76]. Blazhenets et al*.* prospectively assessed Montreal Cognitive Assessment (MoCA) scores and FDG-PET scans of eight COVID-19 patients at the subacute stage and chronic stage (6 months after symptom onset) [11]. Follow-up FDG-PET revealed significantly reduced frontoparietal and temporal hypometabolism which correlated inversely with MoCA performance [11] [Fig. 7—Result of [17]F-FDG-PET group analysis]. Despite significant recoveries of regional neuronal function and cognition, FDG-PET suggested residual impairment in some patients 6 months after COVID-19 infection [11]. These data support the theory that COVID-19 infection leads to underlying systemic inflammation and cortical hypometabolism and illustrate the utility of FDG-PET in assessing the systemic manifestations of COVID-19 infection [3, 11, 76].

While valid concerns related to cost, radiation burden, and exposure time have limited the application of FDG-PET in COVID-19 patients thus far, an overwhelming body of evidence suggests that adopting whole-body FDG-PET/CT for assessment of COVID-19 infection will provide better characterization of the disease and its complications throughout the body [3, 4, 11, 13, 19, 20, 38, 44, 47, 54, 56, 59, 75–78]. We believe whole-body FDG-PET/CT imaging will play a critical role in disclosing the pathophysiology of COVID-19 and assessing the effectiveness of potential treatments. Further study is warranted to confirm the substantial value of whole-body FDG-PET/CT in the assessment, monitoring, and management of patients with COVID-19 infection.

## Conclusion

Evidence suggests that FDG-PET/CT imaging is a highly sensitive modality for the assessment of COVID-19 disease activity with a multitude of potential clinical applications. To date, concerns over cost, access, and undue radiation burden have limited the use of FDG-PET in COVID-19 patients to a small number of individuals where PET imaging was already clinically indicated. With new COVID-19 variants emerging, increasing reports of persistent symptoms, and the effectiveness of specific treatments and vaccinations under active investigation, there is a need for a sensitive, standardized technique for assessing these patients. Chief advantages of FDG-PET/CT include the ability to localize, quantify, and assess local and global inflammatory changes at different points in time. Employing FDG-PET/CT for this purpose may allow for the earliest detection of COVID-19-associated VTE, standardized monitoring of disease progression and response to treatment, and better characterization of the mechanisms underlying persistent infection. Further study is warranted to confirm the substantial value of FDG-PET/CT imaging in the assessment, monitoring, and management of patients with COVID-19 infection.

## Data Availability

No data associated in the manuscript.
